# Exploring the Correlation Between Salt Tolerance and Seed Nutritional Value of Different Quinoa Genotypes Grown Under Saharan Climatic Conditions

**DOI:** 10.3390/plants13223180

**Published:** 2024-11-13

**Authors:** Rahma Goussi, Hatem Ben Jouira, Ouiza Djerroudi Zidane, Jemaa Essemine, Halima Khaled, Salma Nait Mohamed, Malek Smida, Salim Azib, Alia Telli, Arafet Manaa

**Affiliations:** 1 Laboratory of Extremophile Plants, Centre of Biotechnology of Borj Cedria, B.P. 901, Hammam-Lif 2050, Tunisia; 2 Saharan Bio-Resources Laboratory, Safeguarding and Valorization, Kasdi Merbah Ouargla University, Ouargla 30000, Algerias_azib@hotmail.com (S.A.); 3 Shanghai Institute of Plant Physiology and Ecology, 300 Feng Lin Road, Shanghai 200032, China; jemaa@picb.ac.cn; 4 Technical Institute for the Development of Saharan Agriculture (ITDAS), Ouargla 30000, Algeria; 5 Laboratory of Olive Biotechnology, Centre of Biotechnology of Borj Cedria, B.P. 901, Hammam-Lif 2050, Tunisia; 6 Laboratory of Protection of Ecosystem in Arid and Semi-Arid Area, University of KASDI Merbah, PB 511 Ghardaia Road, Ouargla 30000, Algeria

**Keywords:** salt tolerance, variability, nutritional value, SAPs, fatty acids

## Abstract

Quinoa is an annual pseudocereal highly adapted to extreme environments and has become, at this point in time, an extremely popular food due to its exceptional and high nutritional quality. This study aims to investigate the association of quinoa salt tolerance at an early developmental stage with its grain nutritional value under the effect of severe climatic hurdles. The current findings revealed a significant variability between genotypes in salt response attributes at the first development stage, where genotypes Amarilla Sacaca (thereafter, *A. Sacaca*) and QQ57 exhibited high salt tolerance thresholds with a low salt sensitivity index (SI), and a high capacity for Na^+^ sequestration into vacuoles. A significant positive association was detected between salt tolerance degree and yield parameters, saponins (SAPs), and minerals contents, where genotype *A. Sacaca* exhibited the highest SAP content with 3.84 mg.g^−1^ and the highest amounts of K, Ca, P, and Fe. The analysis of fatty acid composition demonstrated a high significant negative correlation between crude fat content and salt SI, and between yield parameters. Despite its low harvest index (HI) and low seed oil content, the salt-tolerant genotype *A. Sacaca* showed a high nutritional quality for seed oil according to its lowest ω6/ω3 ratio (5.6/1) and lowest level of atherogenicity index (AI). The genotype 115R, defined as the most sensitive to salt stress, exhibited a high seed oil quality due to its low lipid peroxidation susceptibility as reflected by its oxidative susceptibility and peroxidizability indexes. The significance of this study includes the identification of valuable quinoa genotypes showing high efficiency in growth and yield under severe stress accompanied by a high nutritional value satisfying the market requirements for healthy, nutritious, and safe food products.

## 1. Introduction

Quinoa (*Chenopodium quinoa* Willd.) is considered as one of the oldest crops, domesticated in southern Peru and Bolivia close to Titicaca Lake, and evidence of human cultivation dates back to between 6000 and 7500 years ago [1,2]. Quinoa is an annual pseudocereal, which was cultivated since ancient times as a staple food of the Inca Empire and played a fundamental role in the diet and culture of the Andean indigenous populations [3]. Quinoa has become an extremely popular food in the last 20 years, especially in Europe and North America, due to its exceptional nutritional quality (compared to cereals) with balanced composition high in protein, amino acid profile, minerals, fatty acids, fibers, and minor compounds such as antioxidants and vitamins [4,5]. Further, due to the absence of gluten, quinoa represents a promising alternative gluten-free food product, especially for celiac patients, and is suitable for those suffering from adverse effects from gluten [6,7,8].

All of the aforementioned compounds can promote health benefits through providing valuable therapeutic properties such as boosting the immune system, assisting in cell repair, with cardiovascular diseases, calcium absorption and transport, increased insulin sensitivity, and even preventing cancer metastasis [5,6,7,9]. Although saponins (SAPs) are considered to be the most anti-nutritional factor in quinoa seeds, several other available studies, dealing with their health-promoting properties, such as those antimicrobial, anticancer, antidiabetic, anti-obesity, antioxidant, and cardio-beneficial, are also of great interest for the pharmaceutical industry [8,10]. Moreover, SAPs can be utilized in various commercial applications in agriculture (e.g., bio-insecticide, bio-fungicide, bio-pesticide), as a natural protection against pathogens and herbivorous organisms [11].

It has also been reported that yield production and the nutritional (and/or anti-nutritional) composition of quinoa seeds vary among ecotypes (defined according to distributional, ecological, agronomic, and morphological criteria) due to the strong genetic variation and due to diverse environmental factors [4,12,13]. There are many “ex situ” conservatories for quinoa in gene banks that use seed properties to conserve germplasm. To date, more than 16,400 accessions and commercial cultivars of quinoa and its wild relatives have been conserved in 59 gene banks across 30 countries, mainly in Bolivia and Peru [14]. The genetic diversity of quinoa was highly reported in several previous studies [14,15,16,17].

The higher level of genetic diversity of quinoa is well correlated with the high degree of adaption and its wide potential to enhance food security through tolerating saline and marginal lands and to alleviate pressure on fertile agricultural soils [18,19]. In fact, quinoa has been cultivated in different ecological zones from sea level in the northwest region of Chile to altitudes over 4000 m above sea level (masl) in the Bolivian Altiplano [20]. Quinoa has exhibited high tolerance to soil salinity [21,22,23], long drought periods [24], frost [25], and high solar radiation [26]. As a halophyte plant, quinoa has been recognized as a climate resilient crop which could be integrated in different marginal agriculture systems, especially saline soils [1,2]. It has been demonstrated that the high degree of quinoa salt tolerance is associated with several mechanisms which are quite complex, including the following: (i) high plant growth performance under high salt concentration; (ii) control of transpiration rate; (iii) Na^+^ sequestration into vacuoles and high K/Na selectivity of potassium versus sodium; (iv) low levels of lipid peroxidation or cellular damage; (v) robust antioxidant defense systems [23].

In addition to its exceptional nutritional quality and value with an excellent balance of essential amino acids, and high content of minerals, vitamins, and fibers, quinoa has great potential in vegetable oil extraction and exploitation, and accordingly it has been considered as an alternative oilseed crop thanks to the quantity and quality of the contained lipid fraction [27]. Thus, quinoa seeds contain between 1.8% and 11% oil, rich in essential fatty acids, which are mainly the polyunsaturated fatty acids omega-6 and omega-3 [28]. Its omega-6 content was significantly higher than those of wheat and rice; additionally, its omega-3 content was about three times that of wheat [29]. Previous studies demonstrated that essential fatty acids have been shown to regulate several cell signaling pathways involved in skin inflammation, dehydration, and tissue degradation [30]. Thereby, quinoa oil can also be used in cosmetics as a moisturizing agent of the skin due to a powerful combination of natural essential fatty acids and vitamin E [31].

Given the importance of quinoa’s nutritional value and its exceptionally high tolerance of drought and salinity and its high potential to offer food security, especially in the face of the predicted future world scenario of increasing salinization and aridity. This study seeks to explore the correlation between salt tolerance at an early development stage and seed nutritional properties of different quinoa genotypes grown under extreme Saharan conditions in South Tunisia as the first study for developing a local quinoa value chain.

## 2. Results and Discussion

### 2.1. Early Salt Tolerance Assessment 

**Figure 1:**
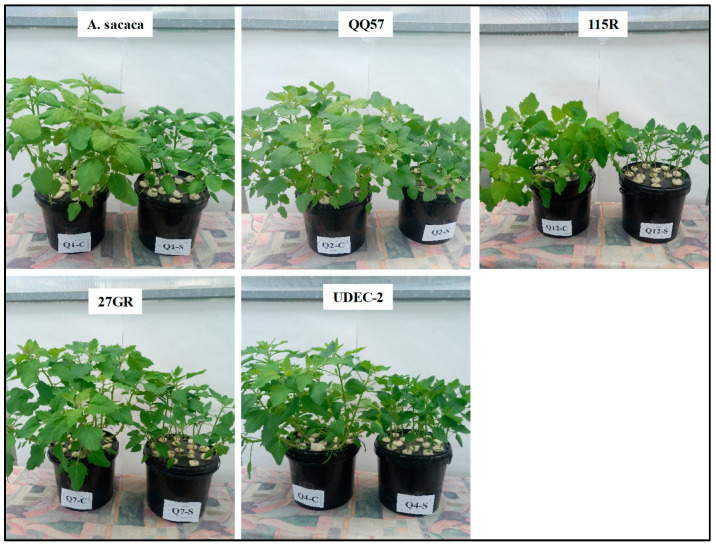
Plant morphological aspects of five quinoa varieties (Amarilla Sacaca, QQ57, 115R, 27GR, and UDEC-2) cultivated under control (C) and sodium chloride (salt, S) treatment (300 mM NaCl). The left plant pot in each image represents the control (C) and the right pot displays the salt-treated plants (S).

**Figure 2:**
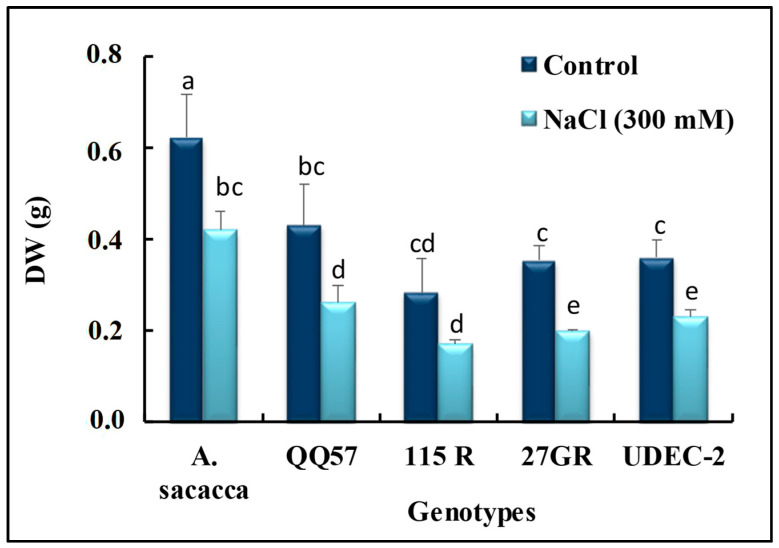
Dry weight (DW) production of five quinoa varieties (Amarilla Sacaca, QQ57, 115R, 27GR, and UDEC-2) cultivated under control (C) and salt treatment (300 mM NaCl). Each data bar of the histogram represents the mean of 6 independent replicates measured on different randomly selected leaves (±SE). The alphabetic letters adjacent to the data bars reflect the significance level of the difference between, in one hand, the quinoa varieties, and, in the other hand, between control and salt-treated plants at a *p*-value < 0.05, based on Duncan’s multiple range tests at 95%.

**Figure 3:**
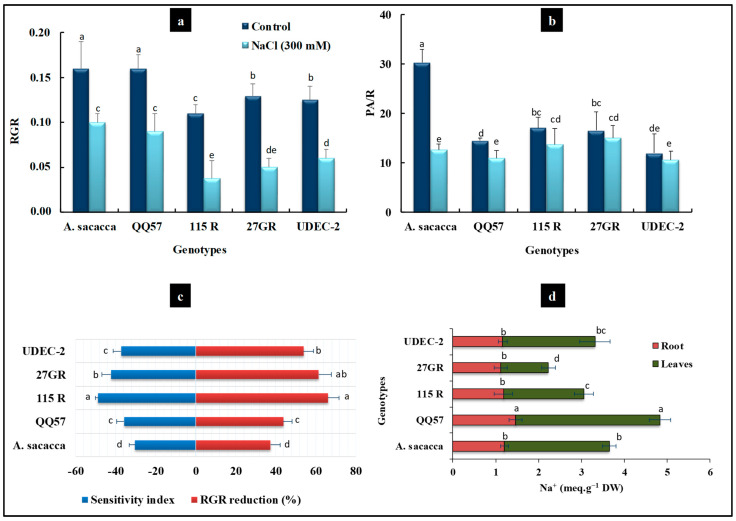
Relative plant growth rate (RGR) (**a**). Shoot/root ratio (**b**). Reduction rate (%) of RGR and sensitivity index SI (**c**). Na^+^ content (**d**) in leaves and roots (meq.g^−1^ DW) of five quinoa varieties cultivated under salt treatment (300 mM NaCl). Each data bar represents the mean of 6 independent replicates (±SE). Means with similar letters are not different at *p* < 0.05 according to Duncan’s multiple range tests at 95%.

**Figure 4:**
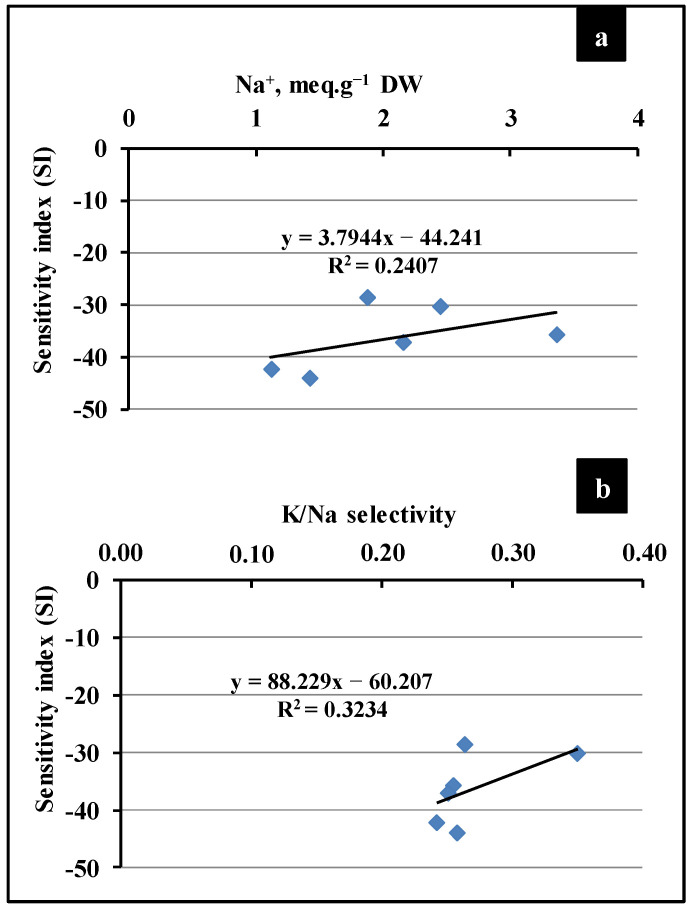
Correlation between sensitivity index (SI) and leaves’ Na^+^ content (**a**) or K/Na selectivity (**b**), of five quinoa varieties cultivated under salt treatment (300 mM NaCl). Data are means of 6 replicates measured on different leaf samples (±SE).

The selection of suitable salt-tolerant quinoa genotypes is an important step to emphasize their adaptation and to examine the effect of severe environmental hurdles on the plant’s development and seed yield [13,32]. In the first step, the present study focuses on assessing the salt stress tolerance degree in five quinoa genotypes at an early growth stage. Our results showed some differences between quinoa genotypes, under control conditions, where *A. Sacaca* showed the highest DW (dry weight), while the other varieties exhibited roughly the same plant growth (Figure 1 and Figure 2). Salt stress treatment significantly decreased plant growth in all varieties without any apparent toxic symptoms such as chlorosis and necrosis (Figure 1). Moreover, the decrease in relative growth rate (RGR) was more prominent in genotypes 115R and 27GR, which was about 66% and 61%, respectively, while for *A. Sacaca* and QQ57 this decrease reached only 37% and 43%, consecutively, in comparison to the control plants (Figure 3a). In this regard, it has been reported that the screening of salt resistant quinoa genotypes requires targeted physiological markers such as salt susceptibility index (SSI) and Na^+^ compartmentalization [21,23]. In the present study, we evaluated the SI based on the differences in DW production measured under stress and non-stress conditions (Figure 3c). Hence, the latter (control) showed that genotype 115R exhibited the highest value of SI with about 48%, while genotypes *A. Sacaca* and QQ57 displayed the lowest SI, which was around 30% and 35%, respectively, indicating that these genotypes reveal a lower biomass reduction under stress conditions. As previously discussed, this plant growth reduction is ascribable to the toxic effects of Na^+^ accumulated in the leaves and roots of all genotypes, where QQ57 sequestrated the highest Na^+^ concentration, suggesting, thus, a high degree of salt tolerance (Figure 3d). Moreover, a positive correlation was observed between leaf Na^+^ accumulation and the SI (r = 0.24; Figure 4a) and K/Na selectivity (r = 0.32; Figure 4b). These observations are in line with the strategy of halophytes known as “salt includer” (the case of genotype QQ57 and *A. Sacaca*) as one of quinoa’s salt tolerance mechanisms, as previously discussed by Derbali et al. [23]. This mechanism includes vacuolar compartmentation or sequestration of toxic ions (especially Na^+^) used for osmotic adjustment [21].

### 2.2. Correlation Between Salt Tolerance Threshold and Agronomic Performance 

As many biological processes in plants can be affected by salinity, salt tolerance mechanisms at early growth stages are not always related to those of the mature stages [33]. Therefore, in this study, we want to explore the correlation between salt tolerance degree at the early developmental stage and agronomic performance at the maturity stage of our tested quinoa genotypes, cultivated outdoors under extreme Saharan conditions in southwest Tunisia. Our experimental site, located near the Chat El Jerid drainage outlet in the southwest Tunisian oasis, was selected as a reference for severe environmental conditions, due to the saline and water-logged soil related to the poor drainage of the oasis, owing to the compacted soil texture and composition [34].

The outdoor-collected results demonstrated a significant variability in different morphological traits between quinoa genotypes. At first, the total growing period of the tested genotypes ranged between 130 (for QQ57, UDEC-2, and 27GR) and 175 days for *A. Sacaca*. As previously discussed, the total growth time was tightly associated with the differences in temperatures, which were mostly found to be strongly influenced by altitude [35,36]. Consistently, our findings are in agreement with those recorded under the same conditions in an oasis of the southern neighboring country Algeria [37].

At the maturity stage, a significant variability in plant growth was observed between quinoa genotypes, where plant height was found to range between 0.6 m for genotype 27GR and 1.5 m for *A. Sacaca*, which was also noticed by its highest produced DW (Table 1). This variability in the plant growth performance physiological trait could be related to the intrinsic genetic difference as corroborated by earlier studies, in which it has been observed that there are obvious differences in plant height between varieties in various Mediterranean regions [36,38]. In the present report, the SI estimate at an early developmental stage showed a positive significant association with the plant growth parameters (DW and plant height) at the maturity stage (Table 2), suggesting a high plant growth performance of the genotype *A. Sacaca* when exposed to moderately high salt stress.

Under salt stress treatment, our data revealed a high variability in quinoa grain yield between the assessed genotypes, and means comparison showed that the highest (38 g) and lowest (13 g) seed yields were observed for *A. Sacaca* (originated from Peru) and 27GR, respectively (Table 1). Our results differed from those documented by Thiam et al. [39], where authors demonstrated that most Peruvian cultivar plants possess a large size and tend to produce a large amount of biomass with few or no seeds despite their well-initiated and established panicles under oasis conditions in the Tinejdad region (Morocco). Moreover, a positive significant association (Table 2) was detected between salt tolerance degree as assessed by (SI) and the different yield parameters (panicle length, panicle weight, and seed yield/plant), emphasizing once again the high plant performance of genotype *A. Sacaca* at the early developmental stage as well as at the maturity stage. This high variability in grain yield and its correlation with quinoa genotype performance at an early developmental stage are certainly correlated with the origin of the cultivars, and the contrasting tested environments [39,40].

For better evaluating seed yield, an HI was calculated based on two defined factors, which are the biomass production and the grain yield (Table 1). The HI is tightly related to the biomass production (depending on photosynthetic efficiency) and its partitioning into the storage organ; however, these processes were shown to be highly sensitive to external environmental stimuli [41]. Our data demonstrated that the genotypes *A. Sacaca* and 27GR showed the lowest HI, which was estimated to equate 0.16 and 0.14, for both genotypes, respectively. While the highest HI was recorded for the genotype UDEC-2 (0.35). In the same trend, *A. Sacaca* and 27GR exhibited the lowest TKW, which was 2.7 and 2.4 g, respectively; however, 115R showed the highest TKW value (3.56 g). This natural variation in quinoa HI was also reported in earlier studies, during which authors demonstrated that the HI could be highly fluctuating, and evolved in the range between 0.06 and 0.87 [39,42].

Furthermore, the correlation matrix analysis (Table 2) does not show any significant correlation between salt tolerance degree (assessed by SI and the yield parameters HI and TKW), demonstrating that stress environmental conditions during the grain filling stage appear to not influence the TKW and HI parameters in entire genotypes. In fact, the variability observed regarding the yield parameters under salt stress is closely associated with the genotypic variation and plasticity of the quinoa pangenome in response to diverse environmental conditions [43]. In fact, the TKW values detected from the tested quinoa genotypes match well and fall into the same range as those previously reported (range between 1.8 and 3.6 g) in Southern Europe [9] and North Africa [39], but they look different relative to the results obtained under the environmental conditions of the Andes (range between 3 and 4.7 g). This reflects a high diversity in this key agronomical trait (TKW) which might be related to the substantial natural genetic variation and/or to the geographical distribution of the studied cultivars [44].

### 2.3. Grain Nutritional and Anti-Nutritional Value

The association of quinoa plant growth performance under stress conditions at an early developmental stage with its grain nutritional value at maturity stage has important implications and guidance in pinpointing valuable and elite genotypes that meet the market demand for healthy, nutritious, and safe food products.

There are more than 3000 quinoa ecotypes whose potential and nutritional values have not yet been well explored outside the Andes and for which a limited number of studies have been performed to assess the seed nutritional quality within severe environments such as the Saharan climate [39,45]. In addition, all the conducted studies confirm that quinoa seeds represent an excellent source of nutritional compounds such as protein, essential amino acids, constituting as well a rich source of vitamins (A, B_2_, E) and minerals (Ca, Fe, Cu, Mg, P, Zn) [18,46]. In the current report, analysis of the elemental composition of quinoa seeds of different genotypes showed that the highest amounts of K, Ca, P, and Fe (1.35, 0.38, 0.29, and 47%, respectively) were detected in *A. Sacaca* (Table 3). 

Interestingly, the genotype 27GR showed the highest Mg content with about 0.28%, and the lowest content was recorded for the 115R genotype with 0.17%. The highest content of iron (Fe) was detected in *A. Sacaca* as well, with a value of 47.3 ppm. Particularly, 115R exhibited the highest potassium, K (1.45%), and sodium, Na (208 ppm), contents, but the lowest Ca (0.24%), P (0.19%), and Fe (29.1 ppm) contents. In this context, our findings are in accordance with earlier reports [47] during which researchers demonstrated significant inter-varietal and intra-varietal differences associated with the genotype × environmental (G × E) interactions’ likelihood. Additionally, it has been reported that the existence of strong inter-varietal and intra-varietal differences in seed mineral concentrations is potentially controlled by the G × E imperative dual relationship in an outdoor experimental site [48].

**Figure 5:**
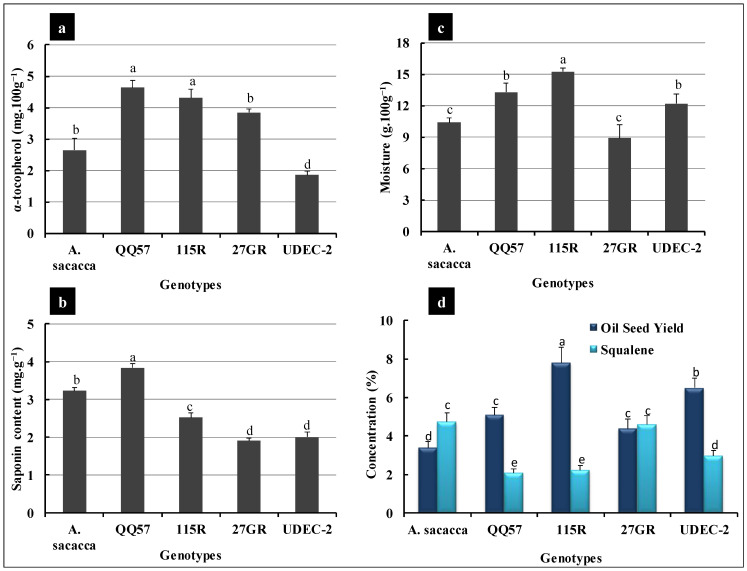
Mean comparison of grain nutritional value in five quinoa varieties (*A. Sacaca*, QQ57, 115R, 27GR, and UDEC-2). α-tocopherol (**a**). Saponins (**b**). Moisture (**c**). Oilseed yield and squalene content (**d**). Each data bar of the histogram represents the mean of 6 independent replicates (±SE). The alphabetic letters adjacent to the data bars reflect the significance level of the difference between quinoa varieties at a *p*-value < 0.05, based on Duncan’s multiple range tests at 95%.

In addition to the high nutritional value of quinoa seeds, SAPs, representing the major anti-nutritional factors, play an important defensive role in plants against pathogens, pests, and herbivores due to their bitter taste [49]. In the present study, a positive significant association was detected between SI and SAP content (Table 2), where genotypes QQ57 and *A. Sacaca* exhibited the highest content, with 3.84 and 3.23 mg.g^−1^, respectively (Figure 5b). However, the lowest SAP content was recorded for genotypes UDEC-2 and 27GR, with about 2 mg.g^−1^ for each of them. Such observations prove once again that the SAP content in quinoa is mainly a genotype- and origin-dependent feature [44,50]. For instance, seeds of varieties belonging to the coastal lowlands ecotype (Chilean origin) are considered bitter, like the Real varieties (known as salares ecotypes) [50,51].

**Figure 6:**
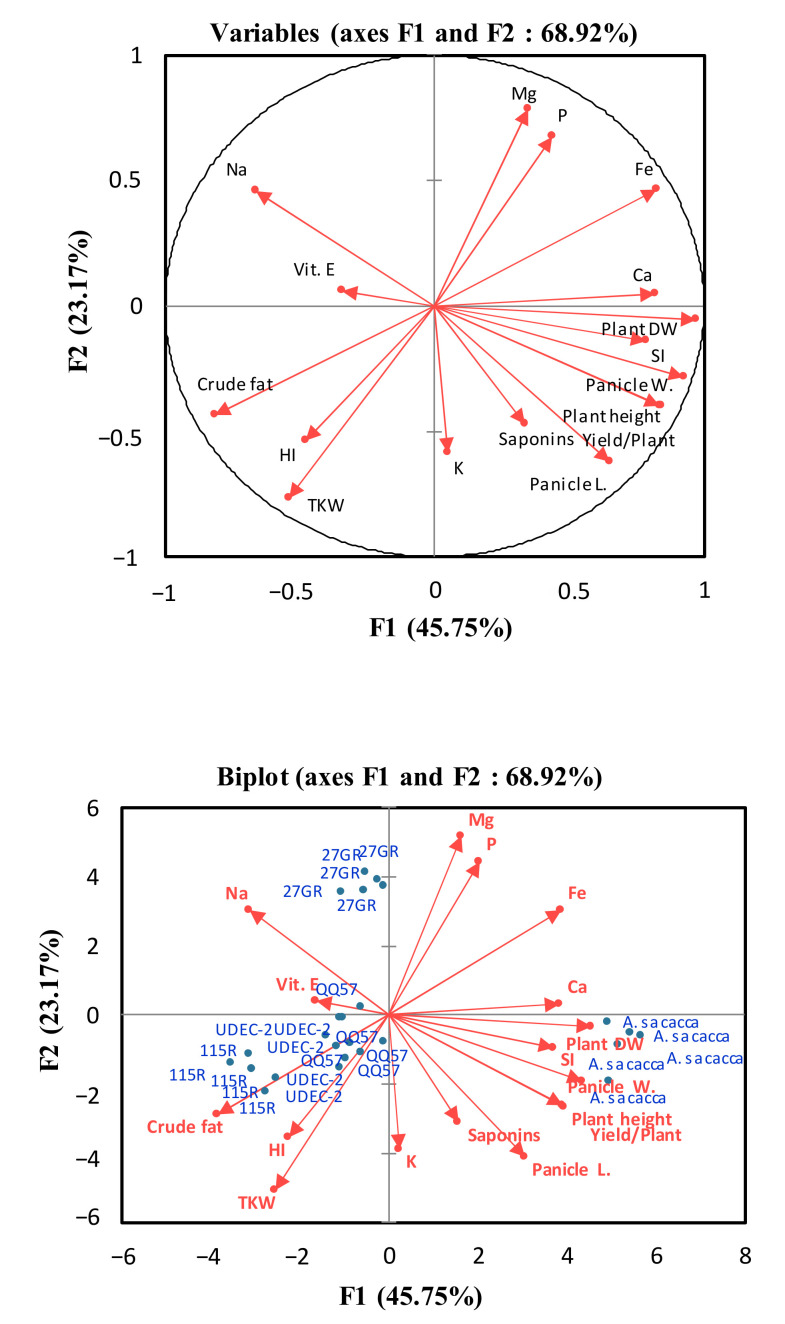
A principal component analysis (PCA) biplot depicting the relationship between the estimated variables (red color) and quinoa genotypes (blue color). F1 on the *x*-axis accounted for 45.7% of the total variability, while F2 explained 23.1% of total variability and is shown on the *y*-axis. Quinoa genotypes: *A. Sacaca*, QQ57, 115R, 27GR, and UDEC-2. Selected variables: sensitivity index, SI; plant height; plant DW; panicle length; panicle weight; seed yield/plant; thousand kernel weight, TKH; harvest index, HI; K, Ca; Mg; P; Fe; Na; vitamin E; saponins; and crude fat content.

A biplot analysis, as a simple and visually descriptive statistical tool, was used in the present study to display, interpret, and explore the G × E interactions (Figure 6). The more positively contributed variables to the F_1_ axis were nutrients (Mg, P, Fe, K, and Ca), plant growth traits (DW and plant height), yield parameters (panicle length, panicle weight, and seed yield/plant), and the SI, suggesting a high plant yield potential and stress tolerance of the *A. Sacaca* genotype (which is similar by way of these parameters). The genotypes 115R and UDEC-2 were at the opposite end of the F_1_ axis and characterized by high crude fat content, HI, and TKW, and low SAP contents. In addition, the mineral traits which contributed positively to the F_2_ axis were Na and vitamin E, suggesting that these components could reflect the salt sensitive attributes of the genotype 27GR within a challenging Saharan climate.

Lipid content is an additional promising and key trait that has been extensively used to assess the nutritional value of quinoa seeds, given that lipids were previously demonstrated to be rich in oil at a level of 3–11% [52,53,54]. Consistently, the crude fat content investigated in this report showed a considerable variability between quinoa varieties, while the average oil content was found to range between 3 and 8% of the total lipid content (Figure 5d). Thus, the highest crude oil content was recorded for 115R and the lowest content was recorded for *A. Sacaca*, at values of 7.8% and 3.4%, respectively (Figure 5d). In addition, Pearson correlation coefficient (PCC) analysis showed a substantial significant negative correlation between crude fat content and SI, and yield parameters (panicle weight, seed yield/plant) (Table 2). Such findings prompt us to suggest that quinoa seed oil content remains steady in all varieties under Saharan conditions and the exploration of FA profiles or abundance could reveal the high nutritional quality of the investigated quinoa genotypes.

### 2.4. Fatty Acid Profile and Its Correlation to Genotype and Healthy Diet

#### 2.4.1. Evaluation of Antioxidant Activity in Quinoa Seed Oil

It has been stressed that quinoa oil has a high quality, which was widely revealed to be associated with the greater antioxidant capacity due to the abundant presence of phenolic compounds (especially, tocopherols) recognized to be involved in ensuring a long shelf life [46]. In addition, α-tocopherol, similarly to vitamin E, acts as an antioxidant at the cell membrane level, thus protecting the fatty acids of the membranes against deleterious effects that can be caused by free radicals [55]. The present study showed a substantial variability in the amount of vitamin E (α-tocopherol) between genotypes, with QQ57 and 115R exhibiting the highest content (4.6 mg.100 g^−1^); on the other hand, UDEC-2 displayed the lowest content with about 1.85 mg.100 g^−1^ (Figure 5a). In this regard, our results are in agreement with previous reports in which authors demonstrated that the amount of vitamin E detected in the six ecotypes of Chilean quinoa ranged between 2.45 and 4.65 mg.100 g^−1^ [56]; but, our results are slightly higher than those for wheat, rice, and barley obtained by Kozioł [57].

Moreover, along with the high amount of vitamin E, quinoa seed oil is rich in several phytosterols, especially squalene, which may serve as an antioxidant compound by quenching singlet oxygen (^1^O_2_), and as protection against the generation of hydrogen peroxide (H_2_O_2_^−^), helping thus to reduce oxidative damage to the human skin [58]. Our essayed quinoa genotypes showed a high amount of squalene, ranging between 2 and 5%, where *A. Sacaca* (4.7%) and 27GR (4.6%) showed the highest squalene content (Figure 5d). This squalene amount was higher than that detected in the selected quinoa line Q5 (ICBA5) recognized and confirmed as the most adapted genotype to marginal environments [48]. This makes the investigated quinoa genotypes, in this study, an invaluable nutritional food for daily consumption.

#### 2.4.2. Fatty Acid Composition Analysis

**Figure 7:**
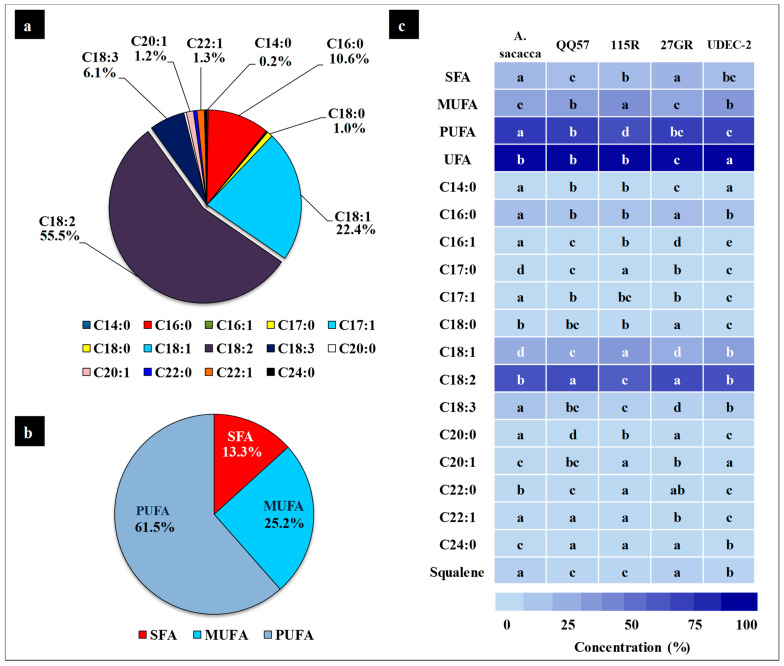
Fatty acid composition of quinoa seeds (**a**). Mean values are shown for each quantified fatty acid, and the values are displayed as an average percentage (%) for five quinoa varieties. The fatty acids detected include the following compounds: myristic_C14:0, palmitic_C16:0, palmitoleic_C16:1, margaric_C17:0, margaroleic_C17:1, stearic_C18:0, oleic_C18:1, linoleic_C18:2, linolenic_C18:3, Arachidic_C20:0, gadoleic_C20:1, behenic_C22:0, erucic_C22:1 and Lignoceric_C24:0. Average content of polyunsaturated fatty acids (PUFA), monounsaturated fatty acids (MUFA), and saturated fatty acids (SFA) in seeds of five quinoa varieties (**b**). Relative concentrations (%) of different fatty acids (**c**) extracted and measured in five quinoa varieties (Amarilla Sacaca, QQ57, 115R, 27GR, and UDEC-2). N = 6 replicates per variety, and different letters indicate significant differences at *p* < 0.05.

The fatty acid composition in five quinoa varieties (*A. Sacaca*, QQ57, 115R, 27GR and UDEC-2) is shown in Figure 7 and Table 4. The most predominant FA is C18:2 (linoleic acid), reaching the highest values in 27GR and 115R with about 58.01 and 57.31%, respectively. This high level of linoleic acid content corroborates the strong antioxidant power of these varieties, as earlier reported [59]. High linoleic acid content has also been considered as a key factor for the high nutritional value of quinoa [60]. The second FA majorly prevailing in quinoa is the oleic acid (C18:1). The latter was significantly higher in 115R (27.61%) compared to the other genotypes (Table 4). The most noteworthy difference in linolenic acid (C18:3) content appeared in genotype *A. Sacaca*, where this genotype shows the highest value, with 9.76% (Figure 7 and Table 4). The detected palmitic acid (C16:0) content was also significantly higher in genotypes 27GR (11.92%) and *A. Sacaca* (11.65%) compared to the three other varieties that are observed to display almost similar values for palmitic acid (~9.7%). In this concern, the recorded results appear to be in line with those of FA profiles reported in quinoa grains cultivated under extreme desert conditions [58]. This variability in major FA concentration among quinoa varieties might be substantially accounted for by the genotype factor, as described by Curti et al. [61]. In the same way, previous studies of Pereira et al. [62] reported no significant difference between black, red, or white seeds, while those of Padmashree and company demonstrated that red quinoa seeds had a lower content of palmitic, linoleic, and linolenic acid than white quinoa seeds [63].

Different minor fatty acids were also detected in our study, including gadoleic acid (C20:1), which was the most abundant one with a total average content of 1.22%, with UDEC-2 and 115R containing the highest levels (with 1.33% and 1.4%, respectively); however, the *A. Sacaca* genotype has the lowest content (1.09%) (Table 4). Similar results were obtained in previous reports with a total average content of C20:1 being about 1.5% [53,64]. When analyzing changes in stearic acid (C18:0) between varieties, it was observed that differences only appeared in genotype 27GR, which showed the highest content (Table 4). The arachidic acid (C20:0) achieved an average value of 0.41%, with small differences between varieties, where 27GR and *A. Sacaca* exhibited the highest amounts.

Interestingly, among the minor detected fatty acids, erucic acid (C22:1) was present in the analyzed quinoa seeds (Table 4). However, FAME analysis showed no considerable differences among quinoa varieties regarding the erucic acid content (with a total average of 1.36%). Furthermore, behenic acid (C22:0) and lignoceric acid (C24:0) were also detected in the present study with the highest level recorded for 111R and 27GR genotypes. Basically, our findings are consistent with an earlier report that used minor fatty acid availability for the discrimination between Andean indigenous crops (quinoa and amaranth varieties) to evaluate the stability and nutritional quality [65].

#### 2.4.3. Fatty Acid Nutritional Quality

Performance of FA content and the nutritional quality of quinoa seeds growing under extreme environment such as desert conditions would give us a clear idea for the selection of valuable genotypes [48]. Moreover, some of FAs could correspond to numerous health benefits, and the excessive amounts could result in health risks associated with different disorders including cardiovascular diseases [66,67]. In our study, the majority of the FAs detected in five quinoa genotypes were: polyunsaturated fatty acids (PUFA), with 61.5% of the total content, followed by monounsaturated fatty acids (MUFA), 25.2%, and saturated fatty acids (SFA), with 13.3% (Figure 7b). In this subject, our results are in line with those found by Tang et al. [62] and Matías et al. [64]. Herein, the obtained data revealed that the lowest PUFA concentration was contained in the variety 115R (56%), lower than that obtained in a previous study which reported a PUFA concentration of 73.4%, 71.7%, and 70.9 for the varieties Titicaca, Achachino, and Hualhuas, respectively [54]. As higher PUFA levels can lead to oil oxidation, reducing the oil quality [68], the lowest PUFA content was displayed by the quinoa varieties studied herein (Table 4), which can be regarded as beneficial events and could be considered as lipid indicators supporting their high nutritional quality. In addition to the antioxidant activity described above for linoleic and oleic acids, the presence of high concentrations of α-linolenic acid, especially in the variety *A. Sacaca* (9.7%) (Table 4), also improves the lipid quality of the quinoa seeds, thus providing a rich source of omega 3 [60].

According to the European Food Safety Authority (EFSA issues advice on existing and emerging food risks), saturated fatty acid (SFA) ingestion should be as low as possible. In the current study, SFA was a less abundant class of FAs in the essayed quinoa varieties, where its highest content (11.9%) was recorded for QQ57 and UDEC-2 genotypes; however, this level remains lower than that previously reported for the *Pasankalla rosada* and *Pandela rosada* varieties, which was 16% [54]. This confirms the high nutritional value of the essayed quinoa genotypes herein. Moreover, earlier reports demonstrated that high polyunsaturated/saturated FA ratio diets hold healthy properties with beneficial effects on cardiovascular risk, improving insulin sensitivity [66,69]. In this study, the highest PUFA/SFA ratio was detected in QQ57 and UDEC-2 varieties at about 5%, and the lowest in 27GR at a value of 3.9% (Table 4).

Further to their energy value, omega-3 and omega-6 provide the essential biological functions that the human body requires for its circadian rhythm, and a lower ratio of omega-6/omega-3 is more convenient in reducing the risk of many chronic diseases [70,71]. In fact, an elevated intake of ω6 results in health risks manifested by different disorders that could be exacerbated, including cardiovascular diseases, cancer, or diabetes [66]. Our data showed that the ω6/ω3 ratio varied depending on the genotype, where the highest ratio was detected for 27GR (14.4:1) followed by QQ57, UDEC 2, and 115R (10:1, on average). The lowest ratio was detected in *A. Sacaca* (5.6:1) which falls close to the recommended daily intake for a healthy diet (5:1–10:1), but remains lower than that previously reported [52,64]. This is further evidence supporting the high nutritional quality of our tested genotypes. The occurrence of ω6/ω3 FAs detected in the *A. Sacaca* genotype is in line with what was recommended to increase the availability and consumption of omega 3-containing foods [72].

The different health promotion indexes calculated and depicted in Table 5 are considered as a good marker to assess the nutritional and medicinal value of various food products [73,74]. The atherogenicity index (AI) relates the presence of lauric (12:0), myristic (14:0), palmitic (16:0), and stearic (18:0) FAs with the occurrence of coronary disease when compared to the effects of MUFAs, especially oleic acid (18:1 n-9) and omega-3 and omega-6 [75]. Our investigated quinoa genotypes exhibited an AI value of 0.14 on average, which was lower than the reference tuna oil (considered as the reference with AI = 0.7) [75,76], indicating a high nutritional value of quinoa grain’s oil in terms of atherogenicity health.

The oxidizability (COX) and oxidative susceptibility (OS) indexes were frequently considered as good markers of the oxidative stability of FAs [73]. The genotype 115R presented the lowest COX value, which was 6.6 (Table 5), showing lower susceptibility to lipid peroxidation [77]. In addition, the values of oxidative susceptibility (OS) determined for our tested quinoa varieties ranged from 2847 for 115R to 3474 for *A. Sacaca*. The registered OS values in this study are in accordance with those previously obtained, where OS ranged between 3087 and 3913 for *Misa quinua* and Titicaca, respectively [54]. Moreover, in comparing the OS recorded by the variety *A. Sacaca* (OS = 3474) with that of olive oil and soybean oil (OS values of 567 and 2987, respectively) reported by Covaci et al. [78], it is plausible to assume that the lipids of quinoa varieties retain high oxidative stability [73].

In this study, the hypocholesterolemic/hypercholesterolemic index (h/H) ranges between 6.7 for 27GR and 8.5 for UDEC 2, and approximately falls to the same level as that obtained by Duarte et al. In, [54] the authors showed, in their report, h/H index values ranging between 5.58 for *Pandela rosada* and 8.5 for Titicaca, reflecting a proportion of hypocholesterolemic FAs more than five times above the values of hypercholesterolemic FAs. It should also be take into account that the hypocholesterolemic FAs reduce the low-density lipoprotein cholesterol (LDL, called bad cholesterol), whereas the hypercholesterolemic FAs increased it [79]. This constitutes evidence once again of the high nutritional value of quinoa seeds’ oil.

The stability of PUFAs and their capacity to be protected from possible oxidation processes could be evaluated according to the peroxidizability index (PI) [80]. Additionally, it has been reported that a high value of PI is considered beneficial for human health [79]. In our study, the PI value ranged between 62 for 115R and 75 for *A. Sacaca*, which shows the lowest ω6/ω3 ratio, lower than previously reported by Duarte et al. [54], where authors reported in their study a PI value ranging between 88 for Misa and 100 for Titicaca. According to previous research [74,80], lipid peroxidation susceptibility increases with increasing PI values, which implies that oil from the studied quinoa seeds, herein, has higher nutritional quality and is more suitable for human consumption than that of, for example, soybean, corn, palm, or sesame.

## 3. Materials and Methods

### 3.1. Plant Material

The plant material used in this study included five genotypes of quinoa (*Chenopodium quinoa* Willd.). These varieties are: QQ57, 115R, 27GR, UDEC-2 (provided by USDA Genebank), and *A. Sacaca*, which was provided by the Technical Institute for the Development of Saharan Agriculture in Algeria (ITDAS). Some characteristics and features of the used seeds are given in Table 6. These genotypes were selected based on a preliminary screening performed in our laboratory.

### 3.2. Lab Experiments for Salt Tolerance Assessment

The salt tolerance levels of quinoa varieties were evaluated early, during the first vegetative stages, using physiological parameters as markers for discrimination. The experiment was conducted under lab-controlled conditions using a hydroponic system for plant cultivation, as previously described in detail for quinoa [23]. Briefly, seeds were surface-sterilized with sodium hypochlorite (20%, *v*/*v*) solution for 5 min, and washed three times with sterile distilled water. Seeds were germinated in a mixture of 2/3 soil, 1/3 sand, and irrigated with distillated water.

Three-week-old seedlings were transferred into a hydroponic medium with half-strength Hewitt’s solution for two weeks. After an acclimation period, the hydroponic medium was replaced by Hewitt’s full-strength nutrient solution, and salinity treatment (300 mM NaCl) was applied (stepwise, increasing by 50 mM every 24 h until the final concentration was reached). The culture was conducted between March and April 2021 at the Centre of Biotechnology of Borj-Cedria (CBBC) in the northeast of Tunisia, (N 36°42′33.25″–E 10°25′37.99″) in a climate-controlled greenhouse with a temperature of 25/18 °C and a relative humidity of 60/70% (day/night), respectively. After 15 days of NaCl treatment, plants were harvested and separated into leaves, stems, and roots, and then were submitted to desiccation treatment in oven at 80 °C for 3 days.

Plant growth was evaluated by measuring the plant fresh (FW) and dry weight (DW). The sensitivity index (SI) was calculated according to Saadallah et al. [81] based on the differences in DW production measured under stress treatment and non-stress (control) conditions as following:

SI _treatment_ = 100 × (DW _treatment_ − DW _control_)/DW _control_

For the measurement of cations, plant material was dried at 80 °C and digested with nitric acid (1% (*v*/*v*) HNO_3_) according to the method of Wolf (1982). K^+^ and Na^+^ were analyzed by flame emission using a spectrophotometer (Eppendorf Geratebau Netherler).

### 3.3. Field Trial Conditions and Experimental Set-Up Design

#### 3.3.1. Climatic Conditions and Soil Properties

The experimental site was located in the southern Tunisian Desert (Douz, Noueil region, 33°30′25.2″ N latitude, 8°50′11.2″ E longitude, 45 m altitude). This location is known for being one of the driest regions in Tunisia with an average monthly temperature of 39.3 °C in July and a minimum average of 5 °C in January (Table 7). The soil texture corresponds to sandy with an alkaline pH (pH = 8.8), with a salinity of about 4.7 mS.cm^−1^. A groundwater source (EC = 3.5 mS.cm^−1^ and pH = 7.4) was used for irrigation based on a drip irrigation system.

#### 3.3.2. Experimental Design and Data Collection at Harvest

The experiment was conducted during two consecutive growing seasons (2021–2022 and 2022–2023). Plant cultivation was based on a randomized complete block design (RCBD) with three replications. Each elementary plot measured 1.5 m^2^ and contained 5 planting lines spaced 25 cm apart. The inter-plant spacing in each line was about 10 cm. For both seasons, seed sowing started on 7 October and harvest between 15 February and 15 March. The seeding rate was about 7 kg.ha^−1^. Fertilizer was applied at sowing (75-120-60; N-P-K), and nitrogen supply (75 kg.h^−1^) was provided at growth stage 12 (extended BBCH, [83]). Plots were regularly inspected for weeds and pests, which were controlled manually.

At physiological maturity and when seed moisture content reached 14%, the selected plants in the middle rows of each plot were harvested. The plant height and dry biomass were recorded, and panicle weight and length were determined for 5 panicles of the same plants. The total seed weight per plant and per plot were measured, and thousand kernel weight (TKW) was also taken.

### 3.4. Seeds Nutritional Value

#### 3.4.1. Mineral Content Assessment

Obtained seeds were ground in a porcelain mortar to a homogenous state. Dried samples (0.5 g) were digested with 6 mL of nitric acid 67% (*w*/*v*), 0.2 mL of hydrofluoric acid 40% (*w*/*v*), and 2 mL H_2_O_2_ 30% (*w*/*v*) in a microwave digestion system under controlled temperature and power conditions. The digested samples were diluted with deionized water to adjust to a final volume of 25 mL. Sodium and potassium concentrations were determined using a Corning 480 flame photometer (Corning Medical and Scientific. Ltd., Halstead, Essex, UK). Mg, Ca, Fe, and P contents were measured by atomic absorption spectrophotometer (SpectrAA 220; Varian, Mulgrave, Victoria, Australia).

#### 3.4.2. Vitamin E Content Estimation

Determination of vitamin E (α-tocopherol) was assayed as earlier described by Backer et al. [84]. Briefly, ground seeds (0.5 g) were homogenized with a mixture of petroleum ether and ethanol (2:1.6 *v*/*v*); the extract was centrifuged at 10,000 rpm for 20 min. To 1 mL of the obtained supernatant, 0.2 mL of 2,2-dipyridyl (2%) was added, mixed thoroughly, and then kept in dark for 5 min. The resulting red product was diluted with 4 mL of distilled water and mixed well. The resulting color in the aqueous layer was measured by reading absorbance at 520 nm using a spectrophotometer. The vitamin E content was calculated using extrapolation on a standard curve made with a known amount of α-tocopherol.

#### 3.4.3. Saponin Quantification

Extraction of total SAPs was performed based on a previous method with some modifications [85]. Samples were extracted with ethanol/water at a ratio of 1:10 (*w*/*v*) for 15 min, with a sonication output amplitude of 75% and under controlled temperature conditions (between 70 and 75 °C). After centrifugation at 3400× *g* for 10 min, the obtained supernatant was dried under a vacuum.

The colorimetric method adapted from Patel and coworkers [86] and Ncube et al. [87] was used for SAPs quantification. Samples were dissolved in 99% ethanol, and then a half volume of ultrapure water was added to the mixture, thus obtaining 10 mg of solution as a final concentration. To a volume of 125 μL of extract, 125 μL of vanillin in ethanol (0.8%, *w*/*v*) and 1.25 mL of sulfuric acid in water (72%, *v*/*v*) were added. The mixture was vortexed, heated at 60 °C for 10 min, and then subjected to ice-cooling for 3 min. Thereafter, the absorbance was measured at 520 nm using a UV–vis spectrophotometer (analytikjena, AG) against the control sample containing methanol. The total SAP content was calculated by extrapolation on a standard curve prepared beforehand with SAPs as the tested substrate (CAS-No 8047-15-2).

#### 3.4.4. Moisture Content

The moisture content of quinoa seeds was measured by oven drying (Memmert UN30) at 105 °C until a constant weight was reached.

#### 3.4.5. Crude Fat Evaluation

The quinoa seed oil was extracted using the Soxhlet extraction system (AOAC, 1990). Seeds were washed thoroughly with tap water, then with deionized water, and finally air-dried at room temperature. About 100 g of ground quinoa powder (0.5 mm) was used for fat extraction by semi-continuous extraction in a Soxhlet apparatus for 5 h, using petroleum as a solvent. The solvent was distilled off and separated at 50 °C with a laboratory-type evaporator. The crude fat was calculated as the percentage (%) of a sample on a dry weight basis.

### 3.5. Fatty Acid Composition

#### 3.5.1. Seed Oil Extraction

Seed samples were ground into a fine powder using a mortar and pestle. Triplicate 1 g samples of the powdered seeds were extracted by the cold method with n-hexane 1:10 *w/v* for 24 h in a cold chamber (at 4 °C), protected from light and with constant stirring. The sample was vacuum-infiltered in order to remove solid material; washed twice with solvent aliquots; and the oils were recovered in a rotary evaporator at 40 °C, protected from light. The obtained oil was stored at −20 °C prior to analysis. The extraction yield was determined by comparing the oil’s weight obtained by cold and Soxhlet extraction.

#### 3.5.2. Gas Chromatography–Flame Ionization Detector (GC–FID)

The fatty acid methyl esters (FAMEs) were prepared as described in the regulation EEC⁄2568⁄91 and amendments. Methyl esters were prepared from seed oil extract, after alkaline trans-esterification treatment, by vigorous shaking of a solution of oil in hexane (0.2 g in 3 mL) with 0.4 mL of 2 N methanolic potassium hydroxide, and analyzed by gas chromatography with a Hewlett–Packard (HP 4890 D) chromatograph (Hewlett–Packard Company, Wilmington, DE, USA) equipped with a flame ionization detector (FID), and a fused silica capillary column (30 m × 0.25 mm i.d. × 0.25 m film thickness; Agilent, Wilmington, DE, USA). An injection volume of 1 µL was used. The temperatures of the injector, the detector, and the oven were held at 220, 250, and 210 °C, respectively. The fatty acid identification was achieved according to their retention times. Seven fatty acids, including C16:0, C16:1, C18:0, C18:1, C18:2, C18:3, and C20:0, were identified.

#### 3.5.3. Gas Chromatography–Mass Spectrometry (GC–MS)

The gas chromatography–mass spectrometry (GC–MS) method was selected for the analysis of the chemical composition of quinoa seeds’ oil. The analysis was carried out on a gas chromatograph HP 6890 (II) coupled to an HP 5972 mass spectrometer (Agilent Technologies, Palo Alto, CA, USA) with electron impact ionization (70 eV). An HP-5MS capillary column (30 m × 0.25 mm, 0.25 μm film thickness; Agilent Technologies, Hewlett-Packard, CA, USA) was employed. The column temperature was programmed to rise from 40 °C to 280 °C at a rate of 5 °C.min^−1^. The carrier gas was helium with a flow rate of 1.2 mL.min^−1^; the split ratio was 60:1. Scan time and mass range were 1 s and 50–550 *m*/*z*, respectively. The injected volume was 1 μL, and the total run time was approximately 45 min.

#### 3.5.4. Compound Identification

The identification of the oil compounds was guided by a comparison of their retention indices, relative to (C8–C22) n-alkanes, to those of the literature or by matching their recorded mass spectra with those stored in the Wiley/NBS mass spectral library and with other published mass spectra [88].

The relative fatty acid composition of each sample was characterized by the mean percentage and standard variation of individual FAs, as well as by FA classes, considering saturated fatty acids (SFA), monounsaturated fatty acids (MUFA), polyunsaturated fatty acids (PUFA), and unsaturated fatty acids (UFA) [76] as follows:

SFA = [C14:0] + [C16:0] + [C18:0] + [C20:0] + [C22:0] + [C24:0]

MUFA = [C16:1] + [C17:1] + [C18:1] + [C20:1] + [C22:1]

PUFA = [C18:2] + [C18:3]

UFA = MUFA + PUFA

#### 3.5.5. Lipid Quality Indexes

The lipid nutritional quality of different quinoa varieties was assessed by several nutritional indexes using data from fatty acid composition (see Section 3.5.4). These indexes can be calculated according to the following equations [54,55,56,57,58,59,60,61,62,63,64,65,66,67,68,69,70,71,72,73,74,75]:

$$\mathrm{Atherogenicity}:AI=\frac{\left[C12:0]+4\;x\;[C14:0]+[C16:0\right]}{\Sigma UFA}$$

$$\mathrm{Thrombogenicity}:\;IT=\frac{\left[C14:0]+[C16:0]+[C18:0\right]}{0.5\;x\;[MUFA]+{0.5\;x\;[PUFA]}_{n-6}{+3\;x\;[PUFA]}_{n-3}+\frac{{\left[PUFA\right]}_{n-3}}{{\left[PUFA\right]}_{n-6}}}$$

$$\mathrm{Oxidizability}:\;COX=\frac{\left[C18:1]+10.3\times [C18:2]+21.6\times [C18:3\right]}{100}$$

Oxidative susceptibility: OS=[MUFA]+45×[C18:2]+100×[C18:3]

$$\mathrm{Hypocholesterolemic}/\mathrm{hypercholesterolemic}\;\mathrm{index}:\;h/H=\frac{\left[C18:1]+[C18:2]+[C18:3\right]}{\left[C14:0]+[C16:0\right]}$$

Peroxidizability: PI=[C18:1]×0.025+[C18:2]+2×[C18:3]

### 3.6. Statistical Analyses

The statistical analyses were performed with the ‘Statistica’ software (version 6.0, Statsoft, 1998). Mean values and standard error (SE) were obtained based on at least 6 replicates for physiological and nutritional value parameters (biomass, seed yield, ions, SAPs, vitamin E) and based on 3 replicates for seed oil extraction and fatty acid (FA) composition. The means were compared using one-way and multivariate analysis of variance (ANOVA) followed by Duncan’s multiple range tests at 95%. A cluster analysis (CA) was performed to discriminate between different genotypes based on FA composition.

## 4. Conclusions

This study was conducted to explore the correlation between stress tolerance thresholds at an early developmental stage and nutritional value (at the maturity stage) of different quinoa genotypes grown under extreme climatic conditions. We demonstrated a significant variability in salt tolerance responses between genotypes, as discussed in reference to morphological, physiological, biochemical, and agronomical traits, where genotype 115R exhibited a high SI to salt stress during first developmental stage, while it revealed a highest crude fat content during the maturity stage. Genotypes *A. Sacaca* and UDEC-2 were well-adapted to salt stress conditions with low SI at an early stage, and high seed yield production. Despite its low HI and high SAP content in comparison to the other varieties, *A. Sacaca* offered a high nutritional quality for seed oil, with an exceptional phytochemical composition that may be accountable for many biological activities.

This underlined variability in nutritional value, agronomic components, and their correlations with the stress tolerance degree at an early developmental stage, was tightly associated with the genotypic variation and plasticity in response to extreme environments, such as under Saharan conditions. This established genotypic variability will contribute to extending the discussion on alternative sustainable and healthier foods and to enhance the economic (agricultural and pharmaceutical industries) value by the sustainable development of new bio products (e.g., quinoa oil for skin, SAPs for bio-insecticides, etc.).

## Figures and Tables

**Table 1:** Plant growth parameters, seed yield production, and the harvest index of different genotypes at maturity stage. Means with similar letters are not different at *p* < 0.05 according to Duncan’s multiple range test at 95%. All values represent the average of two consecutive growing seasons (2021–2022 and 2022–2023).

	Plant Height (cm)	Plant DW(g)	Panicle Length (cm)	Panicle Weight (g)	Seed Yield/ Plant (g)	Thousand Kernel Weight (g)	Harvest Index (HI)
*A. Sacaca*	151.4 ± 5.5 a	241.2 ± 11.5 a	39.8 ± 3.7 a	185.3 ± 7.5 a	38.8 ± 2.8 a	2.78 ± 0.08 c	0.16 c
QQ57	75.6 ± 3.6 c	73.8 ± 3.4 c	28 ± 2.3 b	61.2 ± 3.5 b	19.5 ± 1.9 c	3.13 ± 0.13 b	0.26 b
115R	85.1 ± 3.2 b	60.2 ± 2.8 d	32 ± 3.1 b	58.2 ± 2.6 bc	14.6 ± 0.8 d	3.54 ± 0.20 a	0.24 b
27GR	61.6 ± 3.4 d	95.6 ± 5.8 b	24 ± 2.6 c	46.7 ± 14.1 d	13.1 ± 1.1 d	2.49 ± 0.07 d	0.14 c
UDEC-2	70.9 ± 4.1 c	75.7 ± 3.6 c	30 ± 3.5 b	55.1 ± 3.3 c	26.4 ± 2.2 b	3.56 ± 0.12 ab	0.35 a

**Table 2:** Correlation matrix based on Pearson correlation coefficient of selected parameters (sensitivity index SI, plant height, plant DW, panicle length, panicle weight, seed yield/plant, thousand kernel weight TKH, harvest index (HI), K, Ca, Mg, P, Fe, Na, vitamin E, saponins, and crude fat contents). The colors and values represent the degree of correlation proportional to the correlation coefficient: positive correlations are indicated in blue, negative correlations in red. *, ** Represent difference significance level at *p* < 0.05 and *p* < 0.01, respectively.

	SI																
SI	1	**P. height**															
P. height	0.539 **	**1**	**P. DW**														
P. DW	0.660 **	0.912 **	**1**	**Panicle L.**													
Panicle L.	0.423 *	0.883 **	0.711 **	**1**	**Panicle W.**												
Panicle W.	0.643 **	0.979 **	0.965 **	0.836 **	**1**	**Yield/P.**											
Yield/P.	0.750 **	0.842 **	0.830 **	0.779 **	0.869 **	**1**	**TKW**										
TKW	−0.346	−0.139	−0.480 *	0.150	−0.265	−0.129	**1**	**HI**									
HI	−0.077	−0.339	−0.52 **	−0.087	−0.395	0.026	0.707 **	**1**	**K**								
K	0.031	0.340	0.126	0.463 *	0.239	0.155	0.407 *	0.016	**1**	**Ca**							
Ca	0.687 **	0.622 **	0.781	0.571 **	0.714 **	0.719 **	−0.447 *	−0.327	0.038	**1**	**Mg**						
Mg	0.214	−0.050	0.255	−0.264	0.070	0.028	−0.741 **	−0.454 *	−0.429 *	0.285	**1**	**P**					
P	0.160	0.107	0.391	−0.062	0.200	0.170	−0.668 **	−0.479 *	−0.251	0.432 *	0.680 **	**1**	**Fe**				
Fe	0.557 **	0.538 **	0.791 **	0.208	0.656 **	0.438 *	−0.803 **	−0.745 **	−0.235	0.613 **	0.579 **	0.608 **	**1**	**Na**			
Na	−0.799 **	−0.60 **	−0.56 **	−0.547 **	−0.647 **	−0.716 **	0.052	−0.068	−0.088	−0.388	0.084	0.115	−0.384	**1**	**Vit. E**		
Vit. E	−0.328	−0.237	−0.319	−0.276	−0.284	−0.548 **	0.000	−0.286	0.042	−0.538 **	−0.147	−0.347	−0.093	0.095	**1**	**SAPs**	
SAPs	0.406 *	0.417 *	0.268	0.357	0.399 *	0.321	0.109	−0.007	0.170	−0.063	−0.183	−0.287	0.165	−0.791 **	0.295	**1**	**Crude fat**
Crude fat	−0.706 **	−0.449 *	−0.72 **	−0.167	−0.589 **	−0.462 *	0.792 **	0.615 **	0.249	−0.610 **	−0.623 **	−0.551 **	−0.908 **	0.485 *	0.141	−0.247	**1**

**Table 3:** Mineral seed contents of different quinoa genotypes. Mean ± SD mineral contents are presented as a percentage of seed weight (K, Ca, Mg, and P) or as ppm (Na and Fe). Different letters with each mineral content show statistically significant differences between samples at *p* < 0.05 according to Duncan’s multiple range test at 95%. All values represent the average of two consecutive growing seasons (2021–2022 and 2022–2023).

	K (%)	Ca (%)	Mg (%)	P (%)	Fe (ppm)	Na (ppm)
*A. Sacaca*	1.35 ± 0.06 a	0.38 ± 0.01 a	0.23 ± 0.01 b	0.29 ± 0.03 ab	47.3 ± 2.2 a	141.1 ± 13.2 c
QQ57	1.22 ± 0.07 b	0.26 ± 0.02 c	0.21 ± 0.02 b	0.22 ± 0.02 cd	36.5 ± 1.4 c	146.6 ± 10.1 c
115R	1.45 ± 0.07 a	0.24 ± 0.02 c	0.17 ± 0.01 c	0.19 ± 0.01 d	29.1 ± 2.3 d	208.5 ± 9.5 a
27GR	1.10 ± 0.06 c	0.30 ± 0.01 b	0.28 ± 0.01 a	0.33 ± 0.01 a	42.4 ± 1.4 b	205.3 ± 8.6 a
UDEC-2	1.25 ± 0.06 b	0.31 ± 0.02 b	0.22 ± 0.00 b	0.26 ± 0.02 bc	31.3 ± 2.1 d	183.2 ± 7.4 b

**Table 4:** Fatty acid and squalene content in the seed oil of five quinoa genotypes at maturity stage (in g/100 g seed mass) during the second season (2022–2023). Each value represents the mean ± the standard deviation of five total lipid extractions per genotype. Different letters show statistically significant differences between samples at *p* < 0.05, according to Duncan’s multiple range test at 95%. SFA, saturated fatty acids; MUFA, monounsaturated fatty acids; PUFA, polyunsaturated fatty acids; and UFA, unsaturated fatty acids.

			*A. Sacaca*	QQ57	115R	27GR	UDEC-2
Tetradecanoicacid	Myristicacid	C14:0	0.32 ± 0.03 a	0.22 ± 0.02 b	0.22 ± 0.01 b	0.15 ± 0.01 c	0.28 ± 0.01 a
Hexadecanoicacid	Palmiticacid	C16:0	11.65 ± 0.72 a	9.71 ± 0.31 b	9.70 ± 0.35 b	11.92 ± 1.17 a	9.78 ± 0.33 b
9-*cis*-Hexadecenoic acid	Palmitoleicacid	C16:1	0.16 ± 0.01 a	0.09 ± 0.00 c	0.11 ± 0.00 b	0.07 ± 0.00 d	0.05 ± 0.00 e
Heptadecanoicacid	Margaricacid	C17:0	0.03 ± 0.00 d	0.07 ± 0.00 c	0.15 ± 0.00 a	0.10 ± 0.01 b	0.06 ± 0.00 c
cis-9-Heptadecenoic Acid	Margaroleicacid	C17:1	0.24 ± 0.03 a	0.11 ± 0.01 b	0.08 ± 0.01 bc	0.11 ± 0.02 b	0.06 ± 0.00 c
Octadecanoicacid	Stearicacid	C18:0	0.89 ± 0.08 b	0.73 ± 0.03 bc	0.86 ± 0.11 b	1.80 ± 0.03 a	0.60 ± 0.05 c
omega-9 FA	Oleicacid	C18:1	19.31 ± 0.92 d	21.72 ± 0.95 c	27.61 ± 1.45 a	18.91 ± 1.58 d	24.45 ± 1.45 b
omega-6 FA	Linoleicacid	C18:2	55.01 ± 1.02 b	57.31 ± 1.04 a	50.80 ± 0.44 c	58.01 ± 1.2 a	55.63 ± 0.35 b
omega-3 FA	Linolenicacid	C18:3	9.76 ± 0.35 a	5.24 ± 0.41 bc	5.31 ± 0.22 c	4.01 ± 0.35 d	5.87 ± 0.77 b
Eicosanoicacid	Arachidicacid	C20:0	0.50 ± 0.04 a	0.25 ± 0.05 d	0.43 ± 0.03 b	0.53 ± 0.04 a	0.35 ± 0.02 c
Eicosenoicacid	Gadoleicacid	C20:1	1.09 ± 0.08 c	1.12 ± 0.05 bc	1.40 ± 0.03 a	1.17 ± 0.11 b	1.33 ± 0.01 a
Docosanoicacid	Behenicacid	C22:0	0.65 ± 0.03 b	0.49 ± 0.01 c	0.70 ± 0.05 a	0.67 ± 0.08 ab	0.53 ± 0.02 c
13-Docosenoic acid	Erucic acid	C22:1	1.36 ± 0.10 a	1.40 ± 0.09 a	1.34 ± 0.05 a	1.38 ± 0.09 b	1.35 ± 0.10 c
Tetracosanoicacid	Lignocericacid	C24:0	0.26 ± 0.02 c	0.46 ± 0.05 a	0.49 ± 0.06 a	0.48 ± 0.04 a	0.37 ± 0.01 b
Selected totals and ratios		SFA	14.30 a	11.93 c	12.56 b	15.64 a	11.97 bc
		MUFA	22.15 c	24.43 b	30.54 a	21.42 c	26.95 b
		PUFA	64.77 a	62.56 b	56.11 d	62.02 bc	61.50 c
		UFA	86.92 b	86.99 b	86.65 b	83.45 c	88.45 a
		PUFA/SFA	4.53	5.25	4.47	3.97	5.14
		ω3/ω6	0.18	0.09	0.10	0.07	0.11

**Table 5:** Fatty acid-based nutritional indexes of five quinoa genotypes. The atherogenicity (AI), oxidizability (COX), oxidative susceptibility (OS), hypocholesterolemic index (h/H) and peroxidizability (PI). Different letters, on the same line, show statistically significant differences between samples at *p* < 0.05, according to Duncan’s multiple range tests at 95%.

	*A. Sacaca*	QQ57	115R	27GR	UDEC-2
AI	0.16 a	0.13 b	0.13 b	0.17 a	0.13 b
COX	7.97 a	7.25 ab	6.66 c	7.03 bc	7.24 ab
OS	3474 a	3128 b	2847 c	3033 b	3117 b
h/H	7.02 b	8.49 a	8.44 a	6.71 c	8.55 a
PI	75.01 a	68.34 b	62.11 c	66.51 b	67.98 b

**Table 6:** Characteristics of the tested quinoa genotypes.

ID	Genotypes	Accession	Seed Origin	Seed Source	Seed Color	Vegetative Cycle (Days)
Q1	Amarilla Sacaca	Salare	Peru	ITDAS	Orange	170–180
Q2	QQ57	PI 614885	Biobío, Chile	USDA	White	130–140
Q3	115R	PI 698748	New Mexico, US	USDA	White	150–160
Q4	27GR	PI 698742	New Mexico, US	USDA	Pink	130–140
Q5	UDEC-2	PI 634921	Chile	USDA	White and Brown	130–140

**Table 7:** Geographic distribution, climatic conditions (average 1999–2022), and soil characteristics (depth 0–25 cm) of the study site in the south of Tunisia (Source: Climate-Data.org). The physical and mineralogical properties of soils were measured according to the methods described by Blake and Hartge [82].

Demosite Region				Southern Tunisia			
GIS coordinates				33°30′25.2″ N 8°50′11.2″ E			
Climate region				Oasis (Sahara)			
Average rainfull (mm) *				7.1			
Average temperature (°C) *				21.7			
Max. temperature *				27.8			
Min. temperature *				14.9			
Relative humidity (%)				43.7			
WS (km/day)				176.7			
Soil characteristics							
Texture				sandy soil			
Sand (%)				89.9			
Silt (%)				1.9			
Clay (%)				8.2			
OM (%)				0.24			
pH				8.76			
EC (mS/cm)				4.78			
CaCO_3_ (%) total				<4.0			
CaCO_3_ (%) Active				<3.0			
N (g/kg)				0.1			
C/N				14.4			
Nutrient availability in soil (mg/kg)							
Ca				19,391			
Na				1250			
Mg				443			
K				222			
Fe				4.39			
P				3.15			
Mn				1.10			
Cu				0.40			
Average 1991–2021	Avg. Temperature °C	Min. Temperature °C	Max. Temperature °C	Precipitation (mm)	Humidity (%)	Rainy days (d)	Avg. Sun hours (hours)
Jan.	10.4	5	16.2	15	60	2	8.5
Feb.	12.2	6.3	18.2	7	48	1	9.3
March	16.4	9.8	22.9	12	42	2	10.3
April	20.6	13.7	27.5	9	37	1	11.3
May	24.9	17.7	31.9	4	34	1	12.3
June	29	21.4	36.2	0	31	0	12.8
July	32	24.1	39.3	0	30	0	12.7
Aug.	31.7	24.4	38.6	1	33	0	12.0
Sept.	28.2	22.1	34.4	6	42	1	11.0
Oct.	23.4	17.4	29.3	9	47	1	9.9
Nov.	16.3	10.9	21.9	10	53	2	9.0
Dec.	11.4	6.4	16.9	11	62	2	8.3

Abbreviations: WS, wind speed. OM, organic matter. Max, maximum. Min, minimum. Avg, average. EC, electrical conductivity. N, north. E, east. * represents the yearly average (2021 and 2022).

## Data Availability

Data are contained within the article.
